# LPS-Binding Protein Modulates Acute Renal Fibrosis by Inducing Pericyte-to-Myofibroblast Trans-Differentiation through TLR-4 Signaling

**DOI:** 10.3390/ijms20153682

**Published:** 2019-07-27

**Authors:** Giuseppe Castellano, Alessandra Stasi, Rossana Franzin, Fabio Sallustio, Chiara Divella, Alessandra Spinelli, Giuseppe Stefano Netti, Enrico Fiaccadori, Vincenzo Cantaluppi, Antonio Crovace, Francesco Staffieri, Luca Lacitignola, Giuseppe Grandaliano, Simona Simone, Giovanni Battista Pertosa, Loreto Gesualdo

**Affiliations:** 1 Nephrology, Dialysis and Transplantation Unit, Department of Emergency and Organ Transplantation, University of Bari, 70124 Bari, Italy; 2 Department of Basic Medical Sciences, Neuroscience and Sense Organs, University of Bari, 70124 Bari, Italy; 3 Nephrology, Dialysis and Transplantation Unit, Department of Medical and Surgical Sciences, University of Foggia, 71122 Foggia, Italy; 4 Nephrology Unit, Department of Medicine and Surgery, University of Parma, 43121 Parma, Italy; 5 Department of Translational Medicine, University of Piemonte Orientale, 28100 Novara, Italy; 6 Veterinary Surgery Unit, Department of Emergency and Organ Transplantation, University of Bari, 70010 Bari, Italy

**Keywords:** LPS-binding protein, fibrosis, pericyte, myofibroblast, endotoxemia-induced oliguric kidney injury

## Abstract

During sepsis, the increased synthesis of circulating lipopolysaccharide (LPS)-binding protein (LBP) activates LPS/TLR4 signaling in renal resident cells, leading to acute kidney injury (AKI). Pericytes are the major source of myofibroblasts during chronic kidney disease (CKD), but their involvement in AKI is poorly understood. Here, we investigate the occurrence of pericyte-to-myofibroblast trans-differentiation (PMT) in sepsis-induced AKI. In a swine model of sepsis-induced AKI, PMT was detected within 9 h from LPS injection, as evaluated by the reduction of physiologic PDGFRβ expression and the dysfunctional α-SMA increase in peritubular pericytes. The therapeutic intervention by citrate-based coupled plasma filtration adsorption (CPFA) significantly reduced LBP, TGF-β, and endothelin-1 (ET-1) serum levels, and furthermore preserved PDGFRβ and decreased α-SMA expression in renal biopsies. In vitro, both LPS and septic sera led to PMT with a significant increase in Collagen I synthesis and α-SMA reorganization in contractile fibers by both SMAD2/3-dependent and -independent TGF-β signaling. Interestingly, the removal of LBP from septic plasma inhibited PMT. Finally, LPS-stimulated pericytes secreted LBP and TGF-β and underwent PMT also upon TGF-β receptor-blocking, indicating the crucial pro-fibrotic role of TLR4 signaling. Our data demonstrate that the selective removal of LBP may represent a therapeutic option to prevent PMT and the development of acute renal fibrosis in sepsis-induced AKI.

## 1. Introduction

Sepsis is a multi-organ disease and represents a systemic immune response to a bacterial infection. In critically ill patients, sepsis is the major cause of acute kidney injury (AKI) and is associated with high mortality or risk of chronic kidney disease (CKD) [1]. The pathophysiology of sepsis-induced AKI is complex and characterized by an overwhelming inflammatory response that leads to metabolic dysfunction, tubular damage, and microvascular dysfunction [2,3]. The most common bacteria involved in sepsis-induced AKI are gram-negative, since their outer wall component, named lipopolysaccharide (LPS) or endotoxin, can activate a wide variety of cells through interaction with specific pattern recognition receptors (PRR), such as TLRs (toll-like receptors) [4]. In the kidney, LPS is mainly recognized by TLR4, which is expressed by tubular, endothelial cells [2,5] and pericytes [6]. The cellular response to endotoxin requires a shuttle protein—LPS-binding protein (LBP)—that brings LPS to TLR4 and maximizes intracellular signaling [7,8,9,10,11,12]. 

Renal pericytes are a large population of resident stromal cells lining the peritubular capillaries that stabilize the endothelium. Recently, Heng Zeng et al. evaluated the critical role of capillary pericytes in sepsis-associated vascular destabilization and leakage, which is crucial in the pathogenesis of end-organ dysfunction and septic shock [13,14]. Indeed, sepsis may cause microvascular hyper-permeability via disruption of pericyte/endothelial cell (EC) interactions. Furthermore, renal pericytes represent a major source of the pathological extracellular matrix [6]. Although pericytes do not have specific markers, the receptor tyrosin kinase PDGFRβ is considered a constitutive marker for renal pericyte isolation and characterization [15,16]. It has been extensively shown that after injury, PDGFRβ^+^ pericytes are able to detach from the endothelium, and after migration and differentiation into α-SMA^+^ myofibroblasts, may lead to interstitial fibrosis [17,18,19,20,21].

Renal fibrosis is the common, final process directed to repair tissue injury; however during sepsis-induced AKI overwhelming and persistent inflammation can lead to renal AKI. Despite recent developments in understanding the immunopathology of sepsis, therapeutic advances have been slow. Further protective therapies are based on the concept that increased levels of pro-inflammatory mediators or endotoxin are associated with the development of AKI, whereby their elimination can prevent sepsis-induced AKI. Indeed, elimination of cytokines and endotoxin is feasible by purification of blood in extracorporeal circuit, through a device (membrane, sorbent) where solute (toxins, cytokines) and fluid can be removed [22,23]. In a previous study, we observed a protective effect of coupled plasma filtration adsorption (CPFA) treatment on EC dysfunction and renal fibrosis; we demonstrated that this beneficial effect was due to the clearance of LBP, a soluble carrier of LPS [11,12]. 

Here, we investigate the involvement of LBP and TLR4 signalling in pericyte activation. We demonstrate the occurrence of pericyte-to-myofibroblast trans-differentiation (PMT) in a swine model of LPS-induced oliguric kidney. We elucidate that PMT is regulated by the cross-talk between TLR4 and TGF-β signaling and is mediated by common effectors (as SMADs proteins). We also show that CPFA treatment reduces PMT through a mechanism mediated by LBP removal, which might represent a potential strategy to prevent the occurrence of early fibrosis in patients with sepsis-induced AKI.

## 2. Results

### 2.1. Acute Induction of PMT in Endotoxemia-Induced Oliguric Kidney Injury

**Figure 1:**
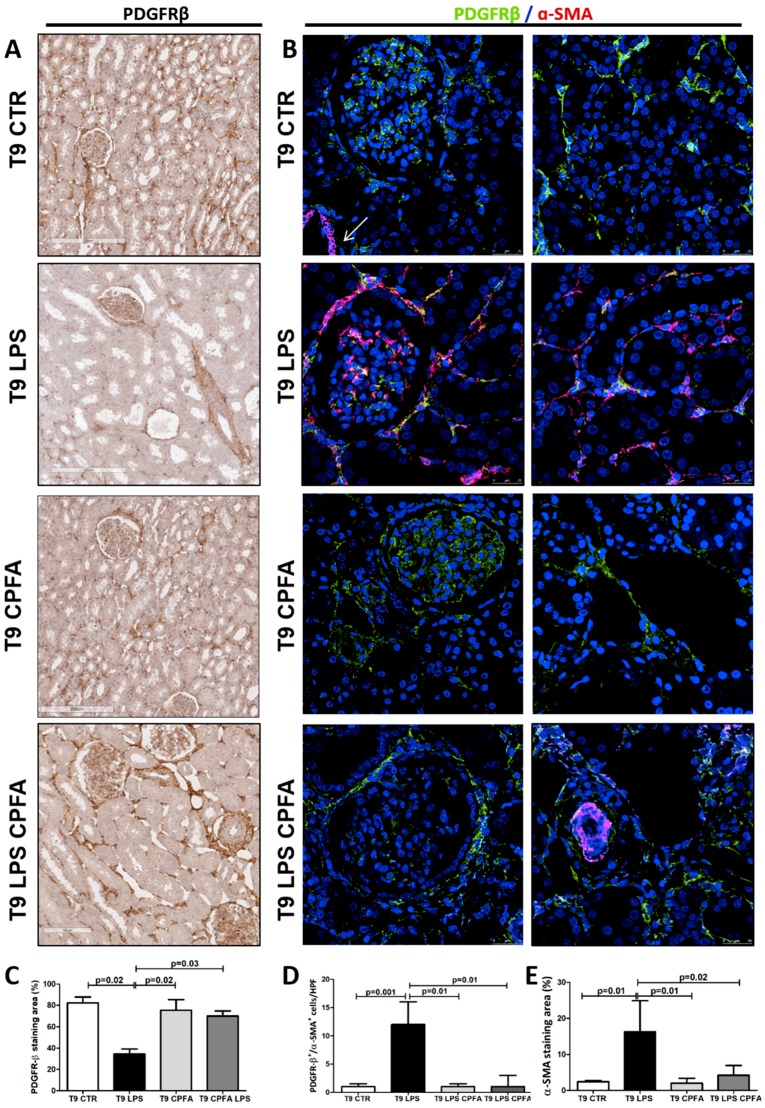
CPFA inhibited LPS-induced PDGFRβ down-regulation and pericyte-to-myofibroblast trans-differentiation (PMT) in endotoxemic pigs. (**A**) IHC (Immunohistochemistry) revealed a strong decrease in PDGFRβ expression at perivascular level after 9 h from LPS infusion (T9 LPS) compared to control (T9 CTR) and CPFA-treated healthy pigs (T9 CPFA). Renal biopsies of endotoxemic animals after CPFA treatment showed a preservation of PDGFRβ^+^ cells (T9 LPS CPFA) Magnification 10x. (**B**) Pericytes were double-stained for PDGFRβ (green) and α-SMA marker (red) to further demonstrate the occurrence of PMT. In the interstitium of T9 CTR and T9 CPFA pigs, PDGFRβ^+^/α-SMA^+^ cells were rarely detectable. Nine h after LPS infusion, the number of these cells dramatically increased (T9 LPS). CPFA treatment reversed LPS-induced PMT, decreasing the number of these transitioning cells (T9 LPS CPFA) Magnification 630×. The fluorescent dye To-pro 3 was used to counterstain nuclei (blue)Quantitative analyses of PDGFRβ (**C**), PDGFRβ/ α-SMA double positive cells (**D**) and α-SMA staining (**E**) were obtained as described in the Methods section and expressed as median ± interquartile range (IQR) of five independent pigs for each group. (**D**) Results are expressed as median ± IQR of the numbers of PDGFRβ^+^/α-SMA^+^ cells/ high-power (×630) fields (HPF) of five independent pigs for each group. Results were statistically analyzed in GraphPad Prism. Statistically significant differences were assessed by the Mann–Whitney test. (**E**) Moreover, α-SMA expression (red-stained area) significantly increased in endotoxemic pigs (T9 LPS) and was reduced by CPFA treatment (T9 LPS CPFA). Magnification 630×

First, we analyzed the activation of renal pericytes in our model of LPS-induced AKI by immunohistochemistry analysis for PDGFRβ. In healthy and CPFA-treated healthy pigs (T9 CTR, T9 CPFA, Figure 1A), PDGFRβ^+^ expression was detected in interstitial peritubular capillaries, in mesangial cells, and Bowman’s capsule. 9 h after LPS infusion, we found a significant reduction of PDGFRβ expression in endotoxemic pigs at peritubular capillary level (T9 LPS) (Figure 1C); on the contrary, PDGFRβ expression of mesangial cells was not significantly down-regulated, as expected [21]. Interestingly, CPFA treatment significantly inhibited PDGFRβ downregulation (Figure 1A, T9 LPS CPFA and Figure 1C).

To investigate whether the PDGFRβ decrease could be associated with occurrence of PMT, we performed a double immunofluorescence for both PDGFRβ and α-SMA marker. In the CTR and CPFA groups (Figure 1B, T9 CTR, and T9 CPFA), PDGFRβ^+^ pericytes were weakly positive for α-SMA, as expected. As shown in Figure 1B and calculated in Figure 1E, α-SMA positivity was predominately found on larger arterial wall (Figure 1B, T9 CTR, white arrow). In the septic pigs, 9 h after LPS infusion (T9 LPS), the phenotype of renal pericytes dramatically changed with a significant increase in α-SMA (Figure 1B, T9 LPS). The co-localization of these two markers (PDGFRβ/α-SMA) was more evident in arterioles, peritubular capillaries and mesangial cells (Figure 1B, T9 LPS), indicating that these cells acquired myofibroblast characteristics. After 9 h of adsorption treatment with CPFA, the number of PDGFRβ^+^/α-SMA^+^ cells in the peritubular capillaries was significantly reduced (Figure 1D), concurrent with the restoring of a physiological cellular phenotype (Figure 1B, T9 LPS CPFA).

### 2.2. LPS-Mediated Early Pericyte-to-Myofibroblast Trans-Differentiation (PMT)

**Figure 2:**
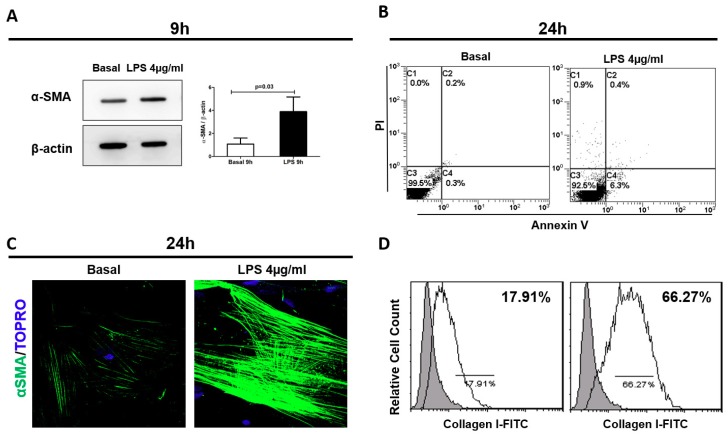
LPS mediated early PMT. Cultured pericytes were incubated with LPS 4 μg/mL, for 9 and 24 h. (**A**) WB analysis revealed a significant increase of α-SMA expression after 9 h of LPS stimulation, compared to basal level. β-actin protein expression was used for normalization. Data are expressed as mean ± standard deviation (SD) of three independent experiments and compared with the Student t test. 24 h of LPS exposure did not affect pericyte viability (**B**) and induced a remodeling of contractile α-SMA-stress fibers (**C**) and a protein increase of Collagen I (**D**). Results are representative of three independent experiments.

To test whether LPS was directly involved in the trans-differentiation of pericytes in myofibroblast, we cultured human pericytes in presence of LPS. A significant increase of α-SMA protein expression was detected after 9 h from LPS stimulation compared to basal condition (LPS:3.9 ± 1.27 vs. basal:1.08 ± 0.5, *p* = 0.03) (Figure 2A).

Moreover, we found that endotoxin exposure did not affect pericyte viability (Figure 2B) and LPS-stimulated pericytes changed their morphology to an elongated and spindle-like cell shape similar to that of fibroblasts. Under immunofluorescence analysis, pericytes showed high α-SMA expression localized in stress fibers, indicating the acquirement of a contractile phenotype (Figure 2C). Finally, flow cytometry analysis revealed that these changes were accompanied by increased Collagen I protein expression (Figure 2D). Collectively, these findings showed that LPS triggered PMT in vitro, indicating the differentiation towards a pro-fibrotic phenotype (Figure 2C,D).

### 2.3. LPS Binding Protein (LBP) Was Critical in LPS-Mediated PMT

**Figure 3:**
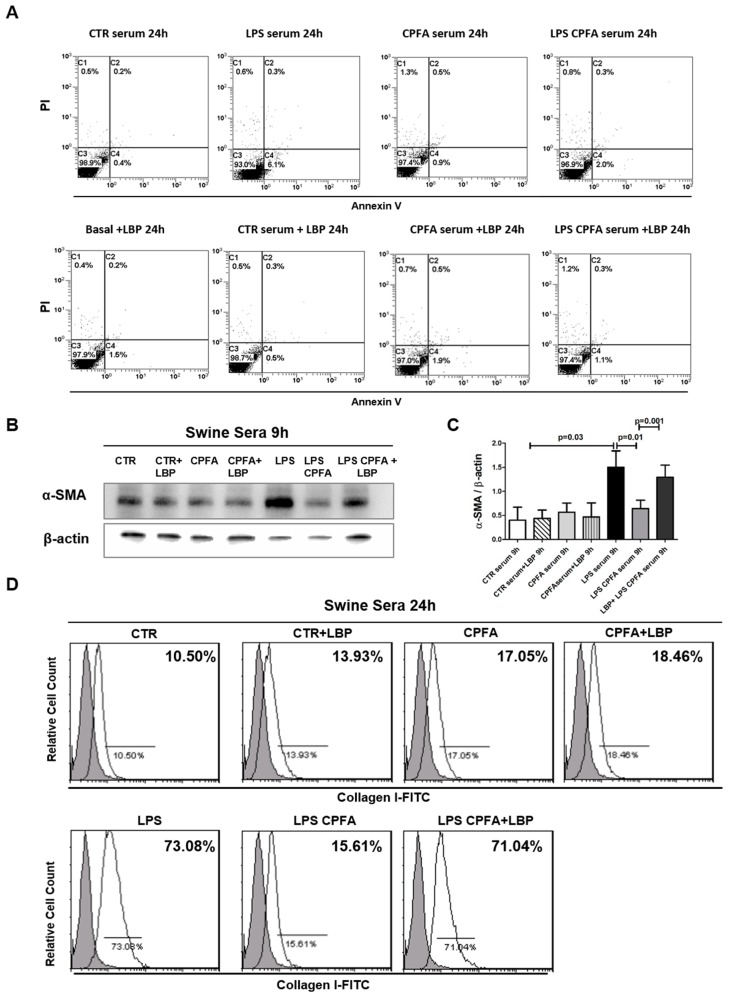
LBP was critical in LPS-mediated early PMT. Cultured pericytes were stimulated with LPS 4 μg/Ml or LBP 9 μg/Ml, or cultured in presence of different swine sera with/without LBP for the indicated time period. (**A**) Pericyte viability was evaluated by flow cytometry analysis (AnnV/PI). Cells did not undergo apoptosis. (**B**,**C**) WB analysis revealed a significant increase of α-SMA expression after 9 h of endotoxemic sera incubation. CPFA-treated septic sera (LPS CPFA) maintained the expression of α-SMA at low level as CPFA-treated healthy sera (CPFA group). Both in CTR and CPFA sera, the addition of exogenous LBP did not increase α-SMA expression. Remarkably, the addition of LBP in CPFA-treated endotoxemic sera (LPS CPFA) induced PMT like the untreated endotoxemic sera. β-actin protein expression was used for normalization. Data were shown as mean ± standard deviation (SD) and compared with the Student t test. (**D**) FACS (Fluorescence Activated Cell Sorting) showed a strong increase of Collagen I after 24 h of endotoxemic sera incubation compared to control. In accordance, CPFA-treated endotoxemic sera maintained Collagen I expression at basal level as CTR and CPFA group. Exogenous LBP supplementation in CTR and CPFA group did not stimulate Collagen I synthesis in pericytes. The LBP addition in LPS CPFA group completely reversed the effects of CPFA treatment, leading to PMT. Results are representative of three independent experiments.

Next, we investigated whether LPS/TLR4 signaling may be critical in mediating PMT during endotoxemia-induced AKI. Recently, we demonstrated in the same animal model that CPFA treatment prevented an LBP serum increase and that the removal of LBP drastically reduced the binding of LPS to TLR4 receptor [11]. We cultured pericytes in the presence of different swine sera for 9 h and 24 h (CTR, CPFA, LPS, and LPS CPFA). Firstly, we examined whether LBP and swine sera could affect pericyte viability. After 24 h of LBP-activation and incubation with swine sera, flow cytometry analysis showed that pericytes did not undergo apoptosis (Figure 3A).

Western blot analysis revealed a significant increase of α-SMA expression after incubation with LPS group sera compared to CTR group sera (data LPS vs. CTR) (Figure 3B,C). CPFA treatment restored the expression of α-SMA at CTR group level in pericytes (LPS CPFA: 0.6 ± 0.21 vs. LPS: 1.02 ± 0.22, *p* = 0.001). Moreover, CPFA-treated healthy sera maintained α-SMA expression at basal level as CTR group.

Flow cytometry analysis also showed a strong increase of Collagen I after 24 h of endotoxemic sera incubation compared to control, indicating that PMT resulted in active contribution to the synthesis of extracellular matrix components (Figure 3D). CPFA-healthy sera did not affect pericyte phenotype. In accordance, CPFA-treated endotoxemic sera maintained Collagen I expression at basal level. 

In order to understand the molecular mechanism involved in the CPFA anti-fibrotic function on human pericyte, we reconstituted the swine group sera with LBP. Both in CTR and CPFA sera, the addition of exogenous LBP did not influence α-SMA and Collagen I expression. Remarkably, the addition of exogenous LBP in sera of LPS CPFA pigs completely reverted the CPFA protective effect. We detected that after LBP sera reconstitution, LPS CPFA group sera re-acquired the capacity to induce PMT in vitro, as shown by the increase of α-SMA and Collagen I protein expression (Figure 3B,D).

### 2.4. TLR4 Signaling Mediates PMT via Enhanced Canonical and Non-Canonical TGF-β Signaling

Next, to identify the signaling involved in PMT and directly activated by LBP/LPS/TLR4 we analyzed the early extracellular signal-regulated kinase 1 (ERK1) phosphorylation, a mediator that is common to TLR4 and pro-fibrotic TGF-β pathways. Regarding TGF-β pathway, we assessed the level of activation of the two different SMAD 2/3-dependent and SMAD2/3- independent signaling. 

**Figure 4:**
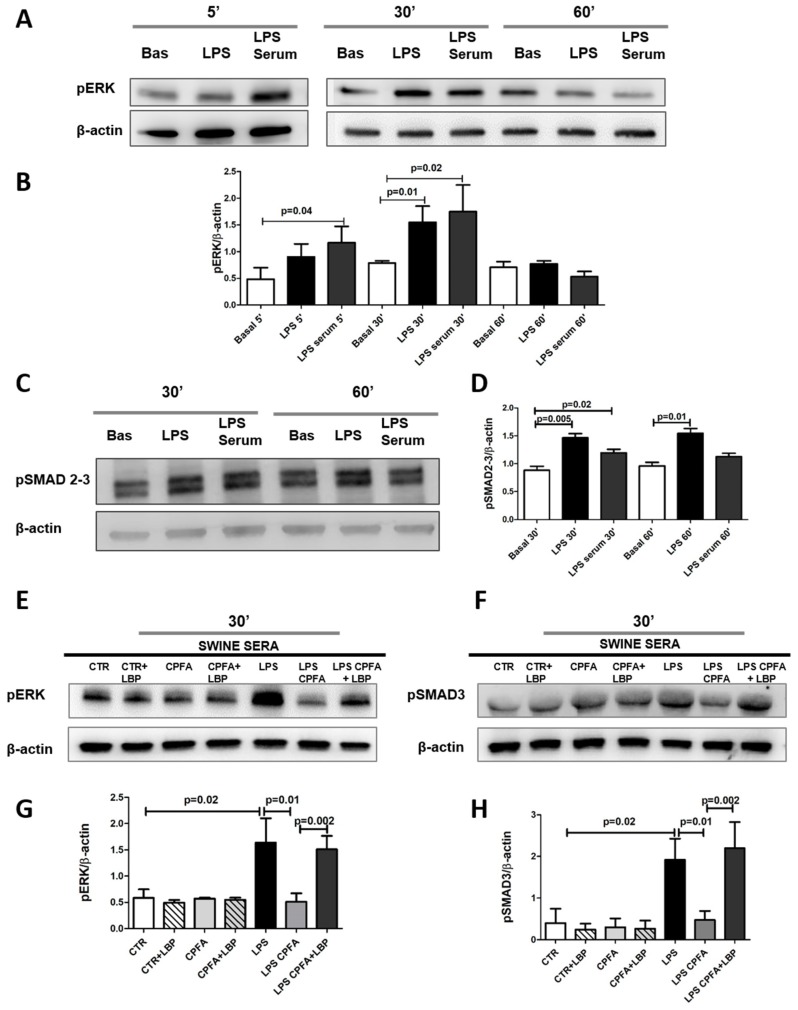
LPS and endotoxemic sera induced PMT by canonical TGF-β-SMAD2/3-dependent and non-canonical TGF-β-SMAD-independent signaling. (**A**–**D**) Pericytes were cultured with LPS or endotoxemic sera for 5, 30, and 60 min. Cell extracts were then used for WB analysis for pERK1 and pSMAD2/3. Both LPS and endotoxemic sera augmented SMAD2/3 and ERK1 phosphorylation, demonstrating the involvement of both canonical TGF-β-SMAD2/3-dependent and non-canonical TGF-β-SMAD-independent signaling. (**E**–**H**) Cultured pericytes were incubated in the presence of 1% of different swine sera for 30 min, with/without LBP (pre-treatment 1 h). CTR and CPFA-treated healthy sera maintained the phosphorylation of SMAD2/3 and ERK1 at basal level. Endotoxemic sera significantly increased phosphorylation of SMAD2/3 and ERK1. CPFA-treated endotoxemic sera reduced the phosphorylation of SMAD2/3 and ERK1 at basal level. The addition of LBP in CTR and CPFA-treated healthy sera did not augment phosphorylation of SMAD2/3 and ERK1. Otherwise, the addition of LBP in LPS CPFA sera reversed CPFA effects, increasing the activation of SMAD2/3 and ERK1. Results are representative of three independent experiments. Data are shown as mean ± standard deviation (SD) and compared with the Student t test.

Pericytes were cultured with or without LPS or sera from LPS group for 5, 30, and 60 min. To analyze the intracellular signaling activated during the incubation, we studied pERK1 and pSMAD2/3 protein expression. Both LPS and endotoxemic sera significantly increased phosphorylation of SMAD2/3 and ERK1, demonstrating the involvement of both canonical SMAD2/3-dependent and non-canonical SMAD2/3-independent signaling, respectively. (Figure 4A–D).

Because we found the maximum increase of pERK1 and pSMAD2/3 level after 30 min of activation by LPS or incubation with septic serum, we decided to further analyze at this time point the potential effect of CPFA treatment on this signaling activation (Figure 4E,F). Compared to LPS group sera, the LPS CPFA group sera led to a significant downregulation of ERK1 and SMAD2/3. In line with the previous findings regarding α-SMA and collagen I protein expression (Figure 3B,D), the restoration of LBP serum significantly increased the phosphorylation of ERK1 and SMAD2/3. Otherwise, the addition of LBP in CTR and CPFA-treated healthy sera did not modify the phosphorylation of SMAD2/3 and ERK1 (Figure 4E,F, quantization in Figure 4G,H). All together, these data indicated that CPFA treatment was able to remove the serum components capable to induce the rapid ERK1 and SMAD2/3 phosphorylation during endotoxemia, and that LBP may be considered a key factor not only in LPS/TLR4 signaling but also in the pro-fibrotic activity of TGF-β pathway.

### 2.5. CPFA Treatment Significantly Decreased Pro-Fibrotic Factors in Endotoxemic Pigs

Several reports have referred to an increase of circulating TGF-β in septic patients [24] and the involvement of Endothelin-1 (ET-1) in the pathogenesis of sepsis [25]. In addition, it is also known that TGF-β can amplify and further enhance LPS signaling [26] and ET-1 promotes the induction of the myofibroblast phenotype from vascular pericytes [27]. 

**Figure 5:**
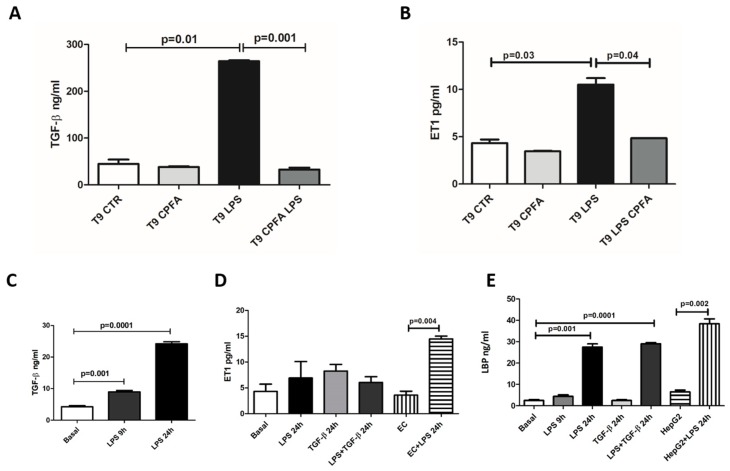
CPFA treatment modulated serum levels of TGF-β and ET-1, and LPS-stimulated pericytes secreted TGF-β and LBP. (**A**,**B**) Serum levels of TGF-β and ET-1 significantly increased after 9 h of LPS infusion compared to healthy pigs. Interestingly, CPFA-treated pigs presented a dramatic reduction in serum TGF-β and ET-1 levels. Data are expressed as median ± IQR of five independent pigs for each group. Statistically significant differences were assessed by the Mann–Whitney test. (**C**–**E**) cultured pericytes were stimulated with LPS and/or TGF-β, and their supernatants were analyzed by ELISA. Human endothelial cells (EC) and human liver hepatocellular cells (HepG2) were used as control positive for ET-1 and LBP synthesis, respectively.(**C**) LPS augmented TGF-β production in pericytes, particularly at 24 h. (**D**) LPS and/or TGF-β stimulation did not increase ET-1 production compared with EC treated with LPS for 24 h (positive control). (**E**) After 24 h from LPS stimulation, pericytes significantly increased LBP synthesis. Stimulation of pericytes with TGF-β alone did not influence LBP production. HepG2 stimulated with LPS for 24 h were used as positive control. Data were shown as mean ± standard deviation (SD) and compared with the Student t test.

In line with these findings, in LPS group sera we found an increase of the TGF-β and ET-1 levels compared to CTR group after 9 h from LPS infusion. Interestingly, CPFA-treated endotoxemic pigs presented a significant reduction in TGF-β and ET-1 serum levels (Figure 5A,B).

In order to clarify the contribution of pericytes to circulating TGF-β, ET-1, and LBP serum release, we evaluated whether LPS exposition could influence their production in culture (Figure 5C–E).

Thereby, we stimulated pericytes with LPS and/or TGF-β for 9 h and 24 h and analyzed the pericyte culture supernatants by ELISA. Interestingly, LPS augmented TGF-β production, particularly at 24 h (LPS 24 h: 24.20 ± 1.51 vs. basal: 4.22 ± 0.88, *p* = 0.001) (Figure 5C). Otherwise, LPS and TGF- β, alone or in combination, did not induce ET-1 production by pericytes.

Because we have already demonstrated [11], in the same animal model, the increase of circulating LBP in endotoxemic pigs, we also evaluated the contribution of pericytes to LBP secretion. 24 h after LPS stimulation, we observed a significant increase of LBP with respect to basal level. Otherwise, TGF-β did not induce LBP synthesis in pericytes.

### 2.6. LBP-LPS Axis-Induced PMT was Characterized by Canonical and Non-Canonical TGF-β Signaling

**Figure 6:**
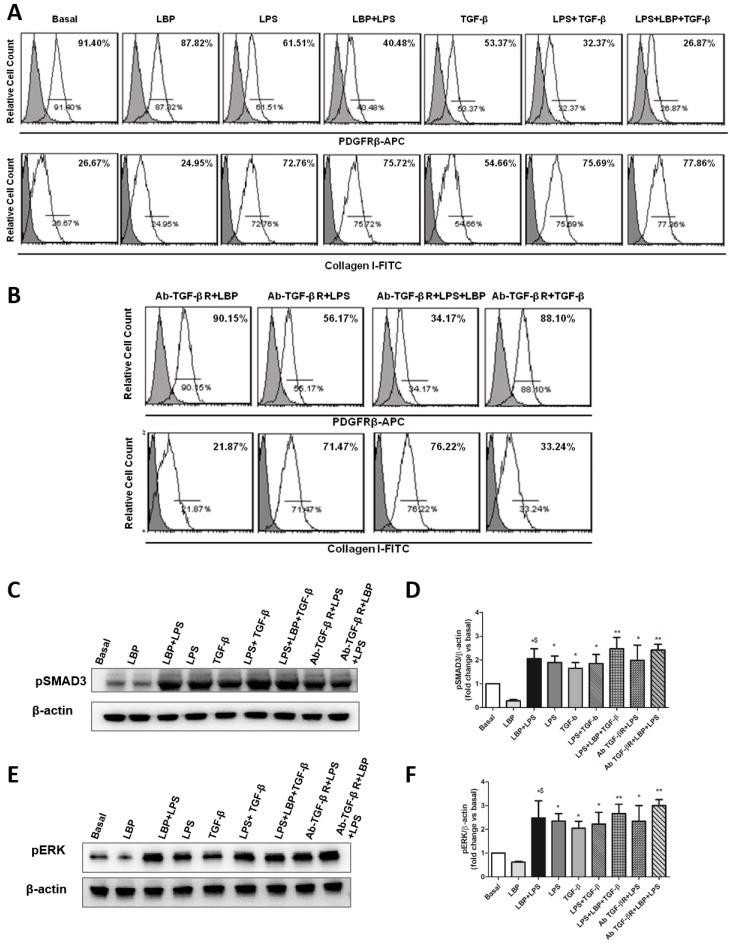
LBP-LPS axis induced PMT upon blocking TGF-βR. (**A**) Cultured pericytes were treated with LBP and/or LPS or TGF- β and with LBP, LPS, and TGF-β mixture for 24 h. LBP alone did not induce phenotypical changes in pericytes. Pericytes treated with LBP and LPS in combination or with LBP, LPS, and TGF-β mixture additively increased Collagen I expression and decreased PDGFRβ marker. (**B**) Pericytes were pretreated with anti-TGF-Βr-specific neutralizing antibody for 1 h followed by LPS and/or LBP stimulation for 24 h. FACS analysis showed that pericytes acquired phenotypic change also upon TGF-βR-blocking. Results are representative of three independent experiments. (**C**–**F**) Pericytes were pretreated with anti-TGF-βR specific neutralizing antibody for 1 h followed by LPS and/or LBP for short stimulation time (30 min). Pretreatment of pericytes with anti-TGF-βR antibody did not reduce LPS and LPS/LBP-induced phosphorylation of both SMAD3 (**C**,**D**) and ERK1/2 (**E**,**F**) at 30 min. Results are representative of three independent experiments. Data are shown as mean ± standard deviation (SD) and compared with the Student t test (**D**,**F**: * *p* < 0.05, ** *p* ≤ 0.005, versus basal level; ^$^ *p* < 0.05 versus LBP).

First, we analyzed the effects of LBP alone or in presence of LPS. In vitro, we observed that LPS-stimulated pericytes contributed to LBP synthesis, which is known to maximize cellular response to endotoxin [11,12,28,29]. Stimulation of pericytes with LBP alone did not induce phenotypical changes in pericytes. Interestingly, pericytes treated with LBP and LPS in combination or with LBP, LPS, and TGF-β mixture additively increased Collagen I expression and decreased PDGFRβ marker more than LPS-stimulated pericytes. These data demonstrate that LBP is a cofactor of LPS that contributes to LPS signaling (Figure 6A). 

Moreover, we observed that LPS-stimulated pericytes are associated with TGF-β secretion, which, in turn, can trigger or enhance PMT [19,21]. Thus, in order to selectively analyze the role of LPS/TLR4 signaling in inducing PMT, we blocked the TGF-β pathway by TGF-βR inhibition (Figure 6B). Pericytes were pre-treated with anti-TGF-βR -specific neutralizing antibody for 1 h followed by LPS and/or LBP stimulation for 24 h. As expected, LBP-stimulated pericytes did not modify their phenotype. Interestingly, we found that LPS or LBP/LPS-stimulated pericytes underwent PMT also following TGF-βR-blocking, as observed by a significant increase of collagen I expression and a decrease of PDGFRβ marker (Figure 6B). These data support the role of LBP-LPS axis in promoting PMT and fibrosis, independently from TGF-β.

Moreover, the anti-TGF-βR neutralizing antibody did not reduce phosphorylation of both ERK1/2 and SMAD3 (Figure 6C–F) mediated by LPS alone or in combination with LBP at 30 min. Stimulation of pericytes with LBP alone did not modify phosphorylation of both ERK1/2 and SMAD3. Collectively, these data indicated that in vitro LPS alone or in combination with LBP can promote both TGF-β canonical and non-canonical pathways, leading to fibrosis and collagen release independently from TGF-β receptor activation.

## 3. Discussion

In this paper, we demonstrated for the first time the occurrence of PMT in a swine model of LPS-induced oliguric kidney. We elucidated that the PMT is regulated by the cross-talk between the TLR-4 and TGF-β signaling and mediated by common intracellular pathways. We also showed that CPFA treatment reduced the PMT through a mechanism mediated by LBP removal [11], which might represent a potential strategy to prevent early fibrosis in patients with sepsis-induced AKI.

Pericytes are mesenchymal-derived cells that stabilize endothelial cells, regulate capillary blood flow, and perform various functions throughout the cellular body [30]. Recent evidence has suggested the prominent role of renal pericytes in scar-forming myofibroblasts generation during CKD [17,18,31] as well as early phases of AKI [21]. Our data demonstrate that renal pericytes are activated also in acute settings such as LPS induced AKI; in vivo and in vitro PMT occurred already after 9 h from LPS activation, with reduced expression of the specific marker PDGFRβ and expression myofibroblast markers α-SMA. Interestingly, in the same swine model of LPS-induced kidney injury, we previously showed that endothelial to mesenchymal transition also contributes to kidney fibrosis [11,12]. Endothelial cells lose their functions and switch from a quiescent to an activated state, acquiring fibroblast phenotype. Taken together, these data indicated pericytes and endothelial cells as the main contributors in the acute induction of renal fibrosis in endotoxemia-induced oliguric kidney injury.

Hyperdinamic renal circulation and an exacerbated inflammatory response without significant evidence of acute tubular necrosis have been extensively characterized in sepsis-induced AKI [32]. In this scenario, the mechanisms involved are attributed to invading microorganisms and their products, known as pathogen-associated molecular patterns (PAMPs), which activate immune cells and renal resident cells by a broad spectrum of PPRs, including TLRs. In particular, LPS, a critical structural component of the outer wall of gram-negative bacteria, is considered the main PAMP and is specifically recognized by TLR-4 [4,33,34]. TLR4 mediates pro-inflammatory and pro-fibrotic pathways, leading to fibroblast accumulation during renal injury [15,35,36,37,38]. Moreover, TLR4-deficient mice were protected from kidney fibrosis with reduced α-SMA protein expression and less tubulointerstitial fibrosis [39]. Recent evidence has demonstrated that TLR4-MyD88-dependent pathway activates not only immune signaling but simultaneously fibrogenesis in pericytes, contributing to matrix deposition and pathology in AKI [6]. Activation of TLR-4 leads to the stimulation of both MyD88-dependent and a MyD88-indipendent pathway that lead to downstream activation of the IKK complex, mitogen-activated protein kinase (MAPK), and phosphatidylinositol 3-kinase (PI3K)/Akt pathways [40]. 

**Figure A1:**
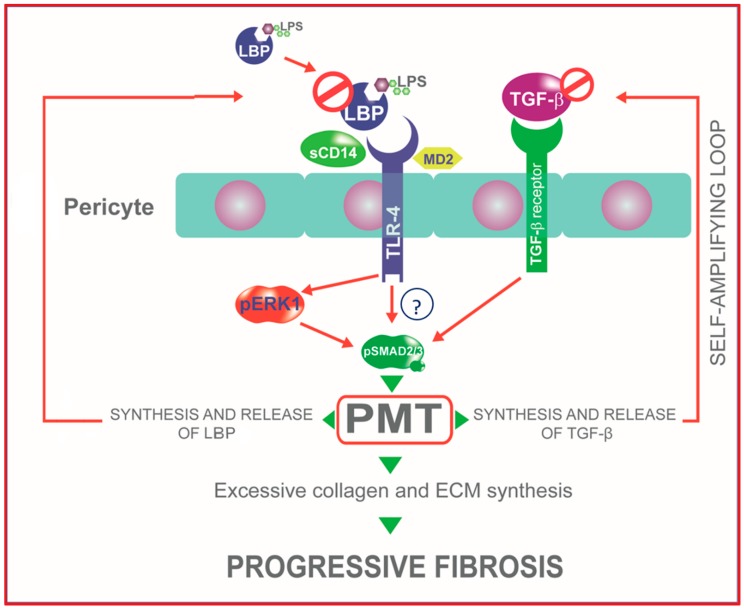
CPFA reduced activation of pericytes and PMT by LBP and TGF-β removal from septic sera. Pericyte TLR4 signaling might contribute to further accumulation of TGF-β and LBP, developing a self-sustaining feed-forward loop that amplifies and maintains renal fibrosis. Disrupting persistent TLR4 signaling activation by the removal of LBP and other molecules as TGF-β represents a potential novel strategy for breaking the cycle of progressive fibrosis.

Our results demonstrated that LPS induced PMT with the acquirement of α-SMA contractile stress fibers and the secretion of ECM products as Collagen I, by triggering TGF-β canonical and non-canonical pathway [41,42,43,44,45,46,47]. Indeed, LPS increased phosphorylation of SMAD2/3 and ERK1, suggesting that acute PMT was induced, respectively, by canonical TGF-β-SMAD-dependent and non-canonical TGF-β-SMAD-independent signaling. (Figure A1)

Moreover, our study showed that LPS can directly induce SMAD2/3 phosphorylation in a TGF-β independent manner. LPS-stimulated pericytes secreted TGF-β and underwent PMT also following TGF-β receptor-blocking, pointing out the pro-fibrotic role of TLR4 signaling. Since LPS may modulate TGF-β synthesis in pericytes, we speculated that LPS/TLR4 signaling contributes to further accumulation of TGF-β, amplifying LPS signaling and developing a self-sustaining positive feedback loop that initiates collagen accumulation and leads to renal fibrosis progression. As LPS and TGF-β additively triggered PMT process in vitro, we suggest that this endotoxin can amplify the responsiveness of pericytes to TGF-β stimulation. Our data are in line with other papers showing that the activation of TLR4 signaling enhanced the sensitivity of fibroblasts to the stimulatory effect of TGF-β, activating SMAD signaling and down-regulating anti-fibrotic antagonist BAMBI [48]. 

Among the different factors involved in TLR-4 signaling, LBP seems to be crucial in enhancing and amplifying cellular response to endotoxin [11,12,28,29]. In vivo, the binding of LPS to TLR-4 requires a carrier protein—LBP—an acute phase protein, synthesized by hepatocytes and released into the bloodstream after gram-negative infection. During the acute inflammatory response, LBP blood levels increase and amplify the host response to infection, even at low concentrations of endotoxin [7,9,10,49]. LBP alone has no effect on the development of tissue injury but serves as a key modulator of cellular and systemic responses to LPS. Moreover, many different types of cells in several organs such as lungs, kidneys, and liver contribute to LBP synthesis, maximizing the local parenchymal inflammation [50]. Here, we showed that pericytes release LBP, as well as TGF-β, in response to LPS. These secretory capacities of pericytes in response to LPS may be particularly important for their contribution to kidney fibrosis.

Moreover, pericytes treated with LBP in combination with LPS and TGF-β significantly amplified PMT process respect to LPS-stimulated pericytes. Our findings are consistent with published data showing that LBP functions as a “biological taxi service” [51] for transporting endotoxin in blood and facilitating LPS binding to TLR-4 maximizing inflammatory response on host cells. 

Accordingly with our previous study, in the same swine model of LPS-induced oliguric kidney injury, endotoxemic pigs with high levels of LBP were prone to inflammation, endothelial dysfunction, early development of fibrosis, and increased risk of mortality [11]. 

**Figure A2:**
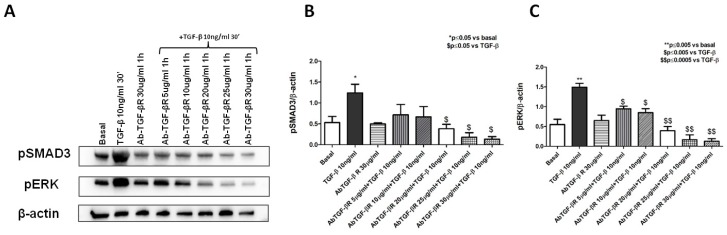
TGF-βR inhibition assay. (**A**–**C**) Pericytes were pre-treated with mouse monoclonal anti- TGF-βR at 5-10-20-25-30 μg/mL for 1 h before the TGF-β (10 ng/mL) exposition for 30 min. Pretreatment of pericytes with anti-TGF-βR antibody at 20 μg/mL is sufficient to significantly reduce TGF-β-induced phosphorylation of SMAD3 and ERK.

Thus, targeting TLR signaling may confer a novel therapeutic strategy for renal fibrosis in early and end-stage renal disease. Until now, soluble receptors and monoclonal antibodies used to block the interaction of LPS and other ligands with TLR-4 did not have any efficacy [52]. Extracorporeal elimination techniques have been proposed as a possible approach for cytokine elimination to improve the clinical conditions of septic patients. Recently, we have demonstrated the beneficial effects of CPFA treatment in LPS-induced oliguric kidney injury. CPFA is a technique of blood purification in which systemic blood circulates through a plasmafilter that separates plasma from whole blood, allowing the non-selective removal of inflammatory mediators through the adsorption resin cartridge [53]. In our previous study, in the same swine model, we demonstrated that the removal of LBP by CPFA, rather than endotoxin, impaired the development of endothelial to mesenchymal transition and prevented the acute development of tubulo-interstitial fibrosis [11]. Accordingly, here we have found that the in vivo removal of LBP strongly reduces LPS binding to TLR4 and the subsequent pericyte activation; moreover, in vitro supplementation of exogenous LBP in treated endotoxemic sera induced the early development of PMT through the activation of TLR-4 signaling and both SMAD dependent and independent TGF-β- signal transduction. On the basis of our results, the in vivo blocking of LBP, rather than endotoxin, may significantly reduce the intracellular signaling regulating pericyte dysfunction and kidney fibrosis (Figure A2). Other studies with knockout mice for LBP are needed to underline the potential benefit of LBP blockade in endotoxemic kidney injury. 

Furthermore, we observed that CPFA treatment was critical in modulating TGF-β serum level in endotoxemic pigs, suggesting the efficacy of adsorption treatments in preventing kidney fibrosis and the subsequent progression to CKD.

ET-1 is also of interest, since it is able to promote the induction of myofibroblast differentiation from vascular pericytes contributing to fibrotic disorders in several organs and tissues. It has been described as an important factor both in renal pathophysiology and kidney diseases. Accordingly, we showed a significant increase of ET-1 in endotoxemic pigs that could contribute to phenotypical changes in pericytes. We also demonstrated for the first time that CPFA treatment reduced ET-1 sera levels, thus regulating the pro-fibrotic process. However, pericytes did not contribute to ET-1 synthesis, and we supposed that other cells like glomerular endothelial cells [27] could be the main source for this factor.

In conclusion, our data suggest that in the early phase of LPS-induced AKI, renal pericytes contribute to renal fibrosis and kidney failure. Extracorporeal treatment by CPFA decrease PMT and might affect fibrosis progression, thereby counteracting the long-term effect of sepsis-induced AKI. 

## 4. Materials and Methods

### 4.1. Animal Model

Animal studies were carried out under protocol approved by Ethical Committee of the Italian Ministry of Health, Prot. N823/2016-PR (2016, approved on 2 September 2016). Briefly, endotoxemia was induced in pig by intravenous infusion of a saline solution containing 300 μg/kg of LPS (lipopolisaccharide membrane of escherichia coli), as described previously [11]. Pigs were divided into four groups: control (CTR, healthy pigs, *n* = 7), CPFA (CPFA-treated healthy pigs, *n* = 7), LPS (endotoxemic pigs, *n* = 7), and LPS CPFA (CPFA-treated endotoxemic pigs, *n* = 7). CTR and CPFA pigs received 10 mL of sterile saline solution.

CPFA treatment was performed for 6 h, as previously described [11]. Animals were sacrified after 9 h from LPS/saline infusion or after 6 h CPFA treatment (T9).

### 4.2. Collection of Samples

Renal biopsies were performed at the start of experimental procedure (T0) and at different intervals from saline or LPS infusion until death (T9). A portion of each biopsy was fixed in buffered formalin (4%) for 12 h and embedded in paraffin by using standard procedures as previously described [11]. Swine sera were collected at T0, at intermediate time points, and at T9 from an arterial blood catheter. 

### 4.3. Cell Culture

Human placental-derived pericytes (PromoCell, Heidelberg, Germany) were grown in Serum-Free Pericyte Growth Medium (PromoCell) at 5% CO2 and 37 °C [21]. Human umbilical vein endothelial cells (HUVEC, EC) and Human Hepato Cancer cells (HepG2) were purchased from American Type Culture Collection (ATCC-LGC Standards S.r.l., Sesto San Giovanni, Milan, Italy). EC and HepG2 were maintained in their recommended medium, EndGro (Merck Millipore, Darmstadt, Germany) and DMEM high-glucose medium supplemented with 10% Fetal Bovine serum (FBS), 100 U/mL penicillin, 0.1 mg/mL streptomycin, 2 mM l-glutamine (Sigma Aldrich, Milan, Italy), respectively. When cells became confluent, they were stimulated with LPS 4 μg/mL (E. Coli O111:B4, Sigma-Aldrich, Milan, Italy), LPS Binding Protein (LBP, HycultBiotech, Uden, The Netherlands) 9 μg/mL [11], and TGF-β1 10 ng/mL (Biovision, San Francisco, CA, USA). Moreover cells were incubated in the presence of 1% of different swine sera with/without LPS Binding Protein (LBP), 9 μg/mL [11], for the indicated time period. All experiments were performed at early P3–P5 passages. For TGF-βR inhibition assay, pericytes were pre-treated with mouse monoclonal anti-TGF-βR (Abcam, Cambridge, UK) at 5–10–20–25–30 μg/mL for 1 h before the TGF-β (10 ng/mL) exposition (Figure A2). The concentration used for TGF-βR blocking before LPS (4 μg/mL) and/or LBP (9 μg/mL) stimulation was 20 μg/mL.

### 4.4. Immunohistochemistry (IHC)

Renal sections underwent deparaffination and heat-mediated antigen retrieval as previously described [11]. Sections were incubated with the primary antibody PDGFRβ (Abcam, Cambridge, MA, USA) and detected by the Peroxidase/DAB Dako Real EnVision Detection System ((Dako, Glostrup, Denmark). Negative controls were obtained by incubation with a control irrelevant antibody. Images were acquired by Aperio ScanScope CS2 device, and signals were analyzed with the ImageScope V12.1.0.5029 (Aperio Technologies, Vista, CA, USA).

### 4.5. Confocal Laser Scanning Microscopy

Swine paraffin-embedded renal sections and cultured pericytes were stained or double stained for α-SMA (Santa Cruz Biotechnologies, Santa Cruz, CA, USA) and PDGFRβ (Abcam, Cambridge, MA, USA) as previously described [21]. All the antibodies cross-reacted with pig tissue. For immunofluorescence microscopy on cultured pericytes, 5 × 10^4^ cells were seeded on a cover slip, grown to 70% confluence, and then stimulated with LPS 4 μg/mL. After stimulation, cells were fixed in 3.7% paraformaldehyde for 5 min. To counterstain nuclei of renal tissue and cells, we used the fluorescent dye TO-PRO-3 (Molecular Probes, Eugene, OR, USA). Image acquisition was performed with confocal microscope Leica TCS SP2 (Leica, Wetzlar, Germany). The number of PDGFRβ+/α-SMA+ cells was quantified in at least 10 high-power (×630) fields (HPF)/sections by two independent observers. The final counts were the mean of the two measures. In no case was interobserver variability higher than 20%.

### 4.6. Detection of Viable and Apoptotic Pericytes by Flow Cytometry Analysis (FACS)

Apoptotic and viable pericytes were evaluated with Annexin V(Ann V)–fluorescein isothiocyanate (FITC) and propidium iodide (PI) according to manufacturers’ instructions (Beckman Coulter, Brea, CA, USA). Three independent experiments were performed. Data were obtained using a FC500 flow cytometer (Beckman Coulter, Brea, CA, USA) and analyzed by Kaluza software.

### 4.7. Immunophenotypic Analysis

After stimulations, pericytes were permeabilized with IntraPrep kit (Instrumentation Laboratory) and incubated with APC-conjugated anti-PDGFRβ (LSBio, Seattle, WA, USA) and FITC-conjugated anti-collagen I (Millipore, Millimarck, Germany) as previously described [21]. Data were analyzed with FC500 (Beckman Coulter, Brea, CA, USA) and Kaluza software. This assay was done in triplicate. The area of positivity was determined by using an isotype-matched mAb, and, in total, 10^4^ events for each sample were acquired. Data were obtained by using a FC500 (Beckman Coulter) flow cytometer and analyzed with Kaluza software. Three independent experiments were performed. 

### 4.8. ELISA for TGF-β, ET-1, and LBP

TGF-β (ELISA; Enzo Life Sciences, Farmingdale, NY, USA) and ET-1 (ELISA; R&D Systems, Minneapolis, MN, USA) levels in swine sera and in cell culture supernatants were measured by a commercially available enzyme-linked immunosorbent assay (ELISA).

LBP levels were revealed in cell culture supernatants were measured by ELISA kit from HycultBiotech (Uden, The Netherlands).

### 4.9. Protein Extraction and Western Blotting

The cell monolayer was rapidly rinsed twice with ice-cold PBS and lysed in RIPA buffer (1 mM PMSF, 5 mM EDTA, 1 mM sodium orthovanadate, 150 mM sodium chloride, 8 μg/mL leupeptin, 1.5% Nonidet P-40, and 20 mM tris-HCl (pH 7.4)) with phosphatase and protease inhibitors. The samples (30 μg of proteins) were separated in 4–15% polyacrylamide gel and then transferred to PVDF membrane (0.2 mM) by Trans-Blot Turbo (BioRad, Hercules, CA, USA). After blocking in BSA at 5%, the membranes were probed with the following primary antibodies: pSMAD2/3 (Abcam), pSMAD3 (Cell Signaling, Danvers, MA, USA), and pERK (Cell Signaling) extracellular signal regulated kinases (ERK) α-SMA (Santa Cruz Biotechnology, Inc.), and then with secondary antibody (hrp-conjugated, Abcam). The same membranes were incubated with mouse monoclonal anti-βactin antibody (1:20,000; Sigma). Immune complexes were detected by the ECL chemiluminescence system (Amersham Pharmacia, Little Chalfont, UK), according to the manufacturer’s instructions. The chemiluminescent blots were acquired by Chemidoc and analyzed using Image J software. Each experiments was repeated three times.

### 4.10. Statistical Analysis

Animal data were expressed as median±interquartile range (IQR) and compared with a Mann–Whitney test. For FACS analysis and western blot, data were shown as mean ± standard deviation (SD) and compared with the Student t test. A *p* value < 0.05 was considered statistically significant. All analyses were performed by using GraphPad Prism 5.0 (GraphPad software, Inc., San Diego, CA, USA).

## Abbreviations

PMT	Pericyte-to-Myofibroblast Trans-differentiation
LBP	LPS Binding Protein
AKI	Acute Kidney Injury
HepG2	Human Hepato Cancer Cells
CKD	Chronic Kidney Disease
CPFA	Citrate-Based Coupled Plasma Filtration Adsorption
EC	Endothelial Cell
WB	Western Blot
TLR-4	Toll Like Receptor-4
ET-1	Endothelin-1
LPS	Lipopolysaccharide
PRR	Pattern Recognition Receptors
Ab	Antibody
TGF-βR	TGF-βReceptor
Ab-TGF-βR	Antibody Anti-TGF-βReceptor
